# Orofacial pain of cardiac origin: Review literature and clinical cases

**DOI:** 10.4317/medoral.17636

**Published:** 2012-02-09

**Authors:** José López-López, Laia Garcia-Vicente, Enric Jané-Salas, Albert Estrugo-Devesa, Eduardo Chimenos-Küstner, Josep Roca-Elias

**Affiliations:** 1 PhD, MD, DDS. Departament of Stomatology. School of Dentistry, University of Barcelona; 2 DDS. Master’s degree in Oral Medicine, University of Barcelona; 3 MD, DDS. Departament of Stomatology. School of Dentistry, University of Barcelona; 4 PhD, MD, DDS. Doctor specialized in Cardiology. Cardiology Service. University Hospital of Bellvitge. School of Medicine, University of Barcelona

## Abstract

The most common types of orofacial pain originate at the dental or periodontal level or in the musculoskeletal structures. However, the patient may present pain in this region even though the source is located elsewhere in the body. One possible source of heterotopic pain is of cardiac origin. Objectives: Report two cases of orofacial pain of cardiac origin and review the clinical cases described in the literature. Study Design: Description of clinical cases and review of clinical cases. Results and conclusions: Nine cases of atypical pain of cardiac origin are recorded, which include 5 females and 4 males. In craniofacial structures, pain of cardiac origin is usually bilateral. At the craniofacial level, the most frequent location described is in the throat and jaw. Pain of cardiac origin is considered atypical due to its location, although roughly 10% of the cases of cardiac ischemia manifest primarily in craniofacial structures. Finally, the differential diagnosis of pain of odontogenic origin must be taken into account with pain of non-odontogenic origin (muscle, psychogenic, neuronal, cardiac, sinus and neurovascular pain) in order to avoid diagnostic errors in the dental practice as well as unnecessary treatments.

** Key words:**Orofacial pain, ischemic heart disease, heterotopic pain, odontalgia.

## Introduction

Ischemic heart disease is one of the major causes of death in adults (1).The clinical description of ischemic heart disease is characterized by substernal pain, which spreads to the shoulders, arms and neck. In some cases, the pain may spread to the jaws and teeth (2,3).The cause of cardiac pain referred to the orofacial region can be explained by convergent mechanisms in the trigeminal complex. On the one hand, visceral cardiac afferences join the somatic sensitive fibers of the upper extremities, of the upper thoracic and cervical region originating pain that usually spreads to the arm. On the other hand, spreading of the pain to the orofacial area is less frequently observed and would be the consequence of converging, at the spinalthalamic tractus, of afferent cardiac fibers with second order trigeminal neurons responsible for innerving the dental sensitivity. However, the most frequent symptom in the pain in the jaw area innerved by the upper cervical roots C2 and C3 (2,4).

The cardiac innervations depend on the afferent sympathetic and parasympathetic nerves (lazy nerve). The majority of the innervations are transferred through the first five thoracic roots, generating that the pain shows in the chest and arms, but not in the face and jaw. The pain does not disappear in patients which were performed a sympathectomia to treat angina, pain. For this reason, it is thought that the lazy nerve plays an important role. The link between the lazy nerve and the trigeminal nucleus explain the pain in the face and jaw (5).

It is known that the most common types of orofacial pain originate at the dental or periodontal sinus level or in musculoskeletal structures. However, the patient may present pain in this region even though the source may be located elsewhere in the body. This type of pain is called “heterotopic”. One possible source of heterotopic pain is pain of cardiac origin (2,4). When these orofacial symptoms occur, unnecessary dental treatment is often performed. There are published clinical cases of patients who have undergone unnecessary dental extractions or have been prescribed analgesic treatments due to the misdiagnosis of temporomandibular disorders, without curing the orofacial pain (4,6,7). This leads to a delay in the diagnosis of infarction or angina, and consequently, a delay in beginning the necessary treatment (2).

## Epidemiology

In developed countries, the misdiagnosis of acute myocardial infarction is observed to occur in 2 to 27% of the cases. A quarter of these errors result in lethal complications for the patient. Patients with atypical symptoms are more likely to be admitted to the hospital than patients with typical symptoms. The key factors for the misdiagnosis are the absence of chest pain and the lack of ST-segment elevation in electrocardiograms (up to 20% in some cases). Therefore, the risk of death for patients with suspected acute myocardial infarction and who do not have chest pain, is three times higher compared to patients seeking care at the emergency room due to chest pain. The risk of death for these patients is eight times higher than that of patients whose chest pain was cured before receiving care at the hospital. There are also studies which show that mortality within one year for patients with symptoms other than chest pain was twice that of patients who suffered chest pain alone (8).

## Pathogenesis

Orofacial pain of cardiac origin is a toothache that occurs spontaneously, usually in relation to exercise; the pain decreases with nitroglycerin tablets and is usually associated with chest pain, anterior neck pain and/or shoulder pain, although it can also manifest as an isolated case. Toothache of cardiac origin may occur as a single clinical manifestation in the oral cavity, affecting adults as well as the elderly and children (9,10). There are also other craniofacial regions where heterotopic pain of cardiac origin can manifest in isolation: the paranasal sinuses, head, mandibular area and the temporomandibular joint region (4,10,11).

Ischemia is a situation caused by oxygen deprivation and the inadequate elimination of metabolites. The mechanism that causes myocardial ischemia is not always the same. In ischemic heart disease, there are two major clinical syndromes: angina pectoris and acute myocardial infarction. Angina is defined as pain, tightness or discomfort -usually in the chest- attributable to transient myocardial ischemia. It is a clinical concept and is diagnosed based on the characteristics and circumstances surrounding the pain. Episodes of angina last between 1 and 10 minutes. Stopping the activity that brought on the pain, resting or taking sublingual nitroglycerin tablets will relieve the pain. A pain lasting less than 30 seconds or continuous pain throughout the day is rarely of cardiac origin. In angina pectoris, there is an increased need for oxygen due to changes in blood pressure and heart rate (exercise and stress in general) in patients with atherosclerotic coronary lesions. In acute myocardial infarction, pain occurs without an apparent cause, suggesting that there has been a spontaneous reduction of oxygen, usually due to coronary thrombosis. In the infarction, the pain usually lasts hours and, unlike angina, myocardial tissue necrosis always occurs, with the possible complications that this entails. Ischemia causes the cellular release of substances such as serotonin, histamine or bradykinin, and allows the accumulation of acid and potassium metabolites. It is believed that one of these substances stimulates the nerve endings and causes pain characteristic of myocardial ischemia (2).

## Clinical conditions

The defining characteristics of angina are thus as follows: location, irradiation, precipitating factors and measures to alleviate the pain. Heart patients normally describe angina pain as a pressure, a weight or a burning sensation, usually located in the retrosternal region and irradiating to the arms, neck or jaw. However, there may be exceptions to this description. Sometimes the pain is described as an unusual discomfort that makes it difficult to breathe, or the pain is located only in the neck, mandible, arms or even the wrists (2,4,12). Odontogenic toothaches are, without a doubt, those which most commonly occur in the oral cavity. Their most significant aspects are presented in  Table 1. The signs and symptoms suggesting that a toothache is not from an odontogenic origin are: not recognizing the pain cause, a burning or pulsatile pain, a pain that does to go into remission or changes, a persistent pain during days, months or years, an spontaneous pain in multiple teeth, a pain that does not go into remission after anesthesic block and the lack of response to an adequate dental treatment. Non-odontogenic pain of a heterotopic origin, which most often occurs in the oral cavity, includes: muscular, neurovascular, neuropathic, sinus, psychogenic and cardiac toothaches ( Table 2).

**Table 1:**
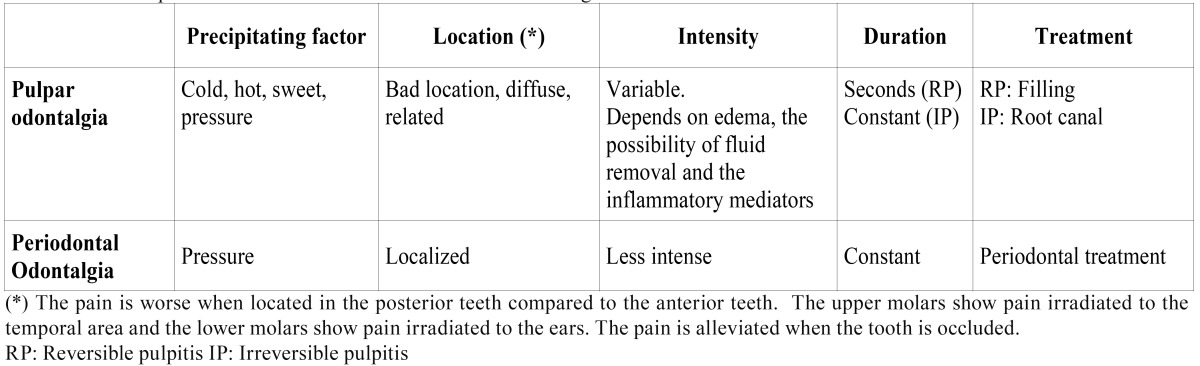
Most important characteristics of toothaches of dental origin.

**Table 2:**
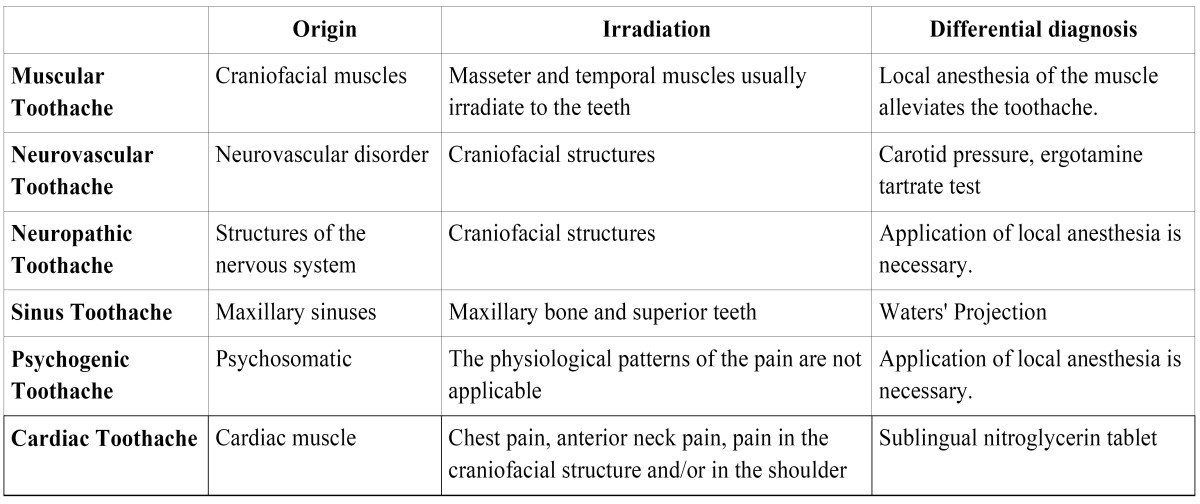
Most significant characteristics of the most common toothaches of non-odontogenic origin.

## Clinical Cases

Clinical case Number 1

This case involves a 54-year-old male with moderate and constant pain located diffusely in the third quadrant, affecting the entire area, including the teeth. The pain has been going on for several days and is not altered by chewing or changes in temperature. The pain has become particularly persistent in the last 24 hours and this has prompted the patient to seek urgent care. The patient has been taking anti-inflammatory medication over the past 15 days due to pain in the neck and back. The patient is being treated for hypertension with Adalat® 10 mg (1 tablet/day) and for type II diabetes with Euglucon® 5 mg (1 tablet/day). There is no relevant medical history. As toxic habits, it is noted that the patient smokes 15 cigarettes a day and is a moderate drinker (2-3 beers/day).

Upon oral examination, we observe that the patient’s mouth is well-maintained, with several fillings; we also note the presence of semi-eruption in tooth 38, but no signs of pericoronaritis and no symptoms. The rest of the examination is normal (dental examination, temporo mandibular joint –TMJ-, masticatory muscles, cervical mobility and cranial nerves). Given the low degree of pain referred by the patient, along with the oral findings observed, the patient undergoes an anesthetic block of the area, which is not conclusive.

Based on the results of the examination, the patient is sent to his family doctor to assess the possibility of conducting a more thorough neurological and cardiological evaluation.

During the follow-up visit, the patient presents a report from the cardiologist which states the diagnosis of unstable angina. Two days after the visit, the patient presented little chest pain associated to a neck and shoulder pain. Because of these manifestations the patient went to a hospital emergency service. The mandibular pain disappeared completely within five days of initiating the treatment prescribed by the cardiologist.

Clinical case number 2

The second case involves a 78-year-old edentulous patient who wears a complete denture on the top and bottom (for more than 10 years) and presents pain felt in the left hemimandible over the last 15 days, concentrated especially on the chin. The pain is constant and dull-with asymptomatic periods-and is associated with pain in the left arm. The patient consulted his family doctor who told him that the mandibular pain may be due to trauma of the denture and he associated the pain in the left arm with the patient’s arthrosis. As relevant medical history, the patient has a long history of type II diabetes, for which he is being treated with Dianben® 850mg (1 tablet/day). The patient has been wearing a full prosthesis on the left knee for the past 2 years.

Upon oral examination, no decubital traumatized areas were noted; the occlusion is well balanced and the pain remained unchanged when chewing objects. The oral origin of the patient’s pain is ruled out and the patient is referred to his family doctor. After performing a cardiological study, the patient was diagnosed with ischemic heart disease.

## Material and Methods

We analyzed the cases presented and carried out a systematic literature review on Medline PubMed using the following keywords: orofacial pain, ischemic heart disease, heterotopic pain, odontalgia, angina pectoris, acute myocardial infarction, mandibular pain. We review 14 articles published between 1987 and 2009, which we reviewed in full text.

## Results

In the scientific literature, there are published clinical cases of patients whose cardiac pathology began as atypical orofacial pain. Of a total of 9 cases reviewed, 4 were males between the age of 63 and 79, and 5 were females between the age of 56 and 76. The pain was only located in the orofacial complex: maxilla, mandible, head, zygomatic arches, submandibular region, neck, temporal area and teeth. In all of the cases except one, the pain irradiated to other areas, such as: the neck, shoulder, infraorbital area, thorax, precordial region, throat and temporal area. The intensity was severe in the 9 cases, occurring spontaneously.

In 2 of these 9 cases, the patient had undergone unnecessary dental treatment due to an initial misdiagnosis-and consequently-without curing the problem. In one of the cases, the patient underwent dental extractions, and in the other case, the patient was diagnosed with a temperomandibular dysfunction as a possible cause of the pain. In 2 of the 9 cases, the onset of the pain was related with physical exercise and in the rest of the cases, the pain occurred spontaneously. The duration of the evolution of the symptoms was only specified in 4 of the clinical cases described and ranged from 3 days to 9 months.

In 6 of the cases, the pain was relieved through the administration of a vasodilator, and in the other 3 patients, the pain was relieved through angioplasty. Following the correct diagnosis of the cardiac involvement and adequate treatment, the bucal symptoms were completely resolved in all of the cases ( Table 3) (2-4,6,7,13,14).

**Table 3:**
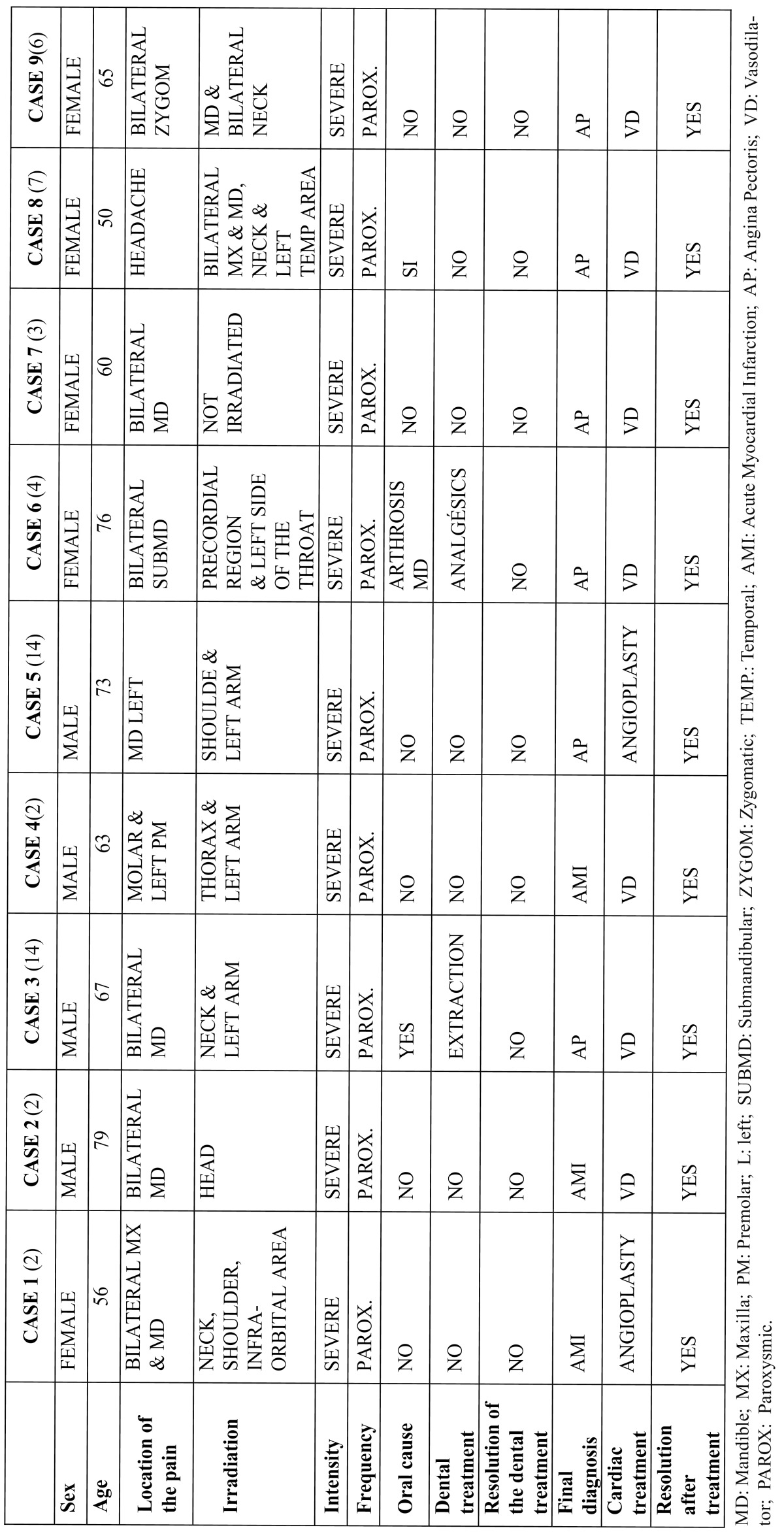
Cases recorded in the literature on heterotopic dental pain.

## Discussion

According to the results of a bibliographic search up to the present 2009, the scientific literature primarily contains isolated clinical cases in patients with orofacial pain of cardiac origin. There was, however, a multicentric study conducted by Kreiner et al. in 2007 (8). The primary objectives of this first study are based on determining the prevalence of orofacial pain on a sample of 186 patients presenting ischemic heart disease as well as describing the location and irradiation of pain. However, are not described other characteristics important to perform a correct and differential clinical diagnostic, such as kind, frequency, intensity, the triggering factors and how to alleviate it. During 2010, Kreiner et al. (15) published another study aiming to differentiate the kind and intensity of toothache in comparison to orofacial pain of cardiac origin. The results concluded that there is no difference in kind between both groups but do exist differences in the description of the pain in relation to its intensity and qualities. Toothache is described as pulsatile and sharp and pain of cardiac origin is described as oppressive and burning. Additionally must be noted that the intensity of pain was higher in patients with toothache than in those with pain of cardiac origin. At intraindividual level the craniofacial pain of cardiac origin is less intense than toothache. However, the intensity increases in locations closer to the heart (15).

The author justifies that these differences relating to intensity and kind of pain are because of the complexity at neurophysiological level of the cerebral complex (15). The explanation of this process is based on the one hand, in certain cortex location responsible for codifying the intensity of visceral pain and in the bilateral cortex locations processing the pain (15,16). On the other hand the convergence of somatic and visceral impulses at the Central Nervous System (CNS), including the trigeminal nucleus (17,18) and the processes of central sensitization (15,19,20).

Pain originating in the heart in craniofacial structures is usually bilateral, whereas odontogenic pain is always unilateral. The most frequent location described for craniofacial structures is in the throat and mandible (8). However, in the literature, we find other orofacial locations where the cardiac pain originates: neck, maxilla, zygomatic arches, head, temporomandibular joint, ears and teeth (4,6-8,15). Due to its location, pain of cardiac origin is considered unusual. However, studies such as those conducted by Kreiner et al. (8) show that for 1 out of every 15 patients who present cardiac isquemia, it manifests in the craniofacial structures. Considering that isquemic cardiopathy is one of the main causes of death among the adult population, there is clearly a clinical underestimate considered to be atypical clinical features, and therefore, this data is significant (7).

Pain of cardiac origin and manifesting in the orofacial area may irradiate to other craniofacial structures (throat, neck, temporal area, head, infraorbital region, maxilla) or to the thorax region (thorax, shoulders, arms).2-4,6-8,14 Odontogenic pain (pulpar or periodontal) can be reflected in structures such as the ears and the temporal area. In this case, if the pain is of cardiac origin, the temporal area coincides with one of the areas mentioned. However, pain of dental origin never refers to the typical areas of precordial pain, such as the thorax, arms and shoulders. According to Kreiner et al. (8) 32% of the patients presented concomitant craniofacial pain in other regions and only 6% presented craniofacial pain as the only symptom during the isquemic episode. Craniofacial pain was predominantly present in females and was the main symptom in both sexes, without the presence of chest pain.

The frequency of the pain in the 9 cases described in the bibliography presents a spontaneous appearance and the intensity is severe in all cases (2-4,6-8,14). However, it is necessary to conduct broader studies and with a larger sample of patients, in order to determine the characteristics of the orofacial pain of cardiac origin, to avoid unnecessary dental treatments such as dental extractions and non-indicated temporomandibular dysfunction therapies, and to not delay the correct diagnosis of heart disease (7,14).

In the oral cavity, when pain is of a pulpar or periodontal odontogenic origin, the cause is detected using direct methods such as the visual method or by complementary techniques such as hot, cold, tapping and other basic tests in order to trigger the painful stimulus.

In isquemic cardiopathy, the painful stimulus is triggered by oxygen deprivation in the coronary arteries; the patient may then suffer angina, if no cellular necrosis of the tissue is present, or may otherwise cause a heart attack. Consequently, the symptoms of the supposed odontalgia will decrease with vasodilators such as nitroglycerine, or with the revascularization of the damaged area, as described in the 9 clinical cases presented above (2-4,6-8,14). The methods for detecting isquemic cardiopathy include three basic pillars: clinical exploration, an electrocardiogram and the markers of myocardial damage. In many cases, the clinical pain -which is different from odontogenic pain- will be accompanied by neurovegetative symptoms such as sweating, vomiting and dizziness.

In the two cases described pain is not very intense, on the contrary to what usually is registered in the literature analyzed in relation to isolated clinic cases (7,14), but it is worth mentioning that toothache is more intense than pain of cardiac origin (15). In addition, the pain is unilateral in both cases, consistent with some other cases recorded (2-4,6,14). No unnecessary dental treatments occurred, in part due to the absence of dental disorders that may confuse the diagnosis.

Finally, all of the articles reviewed conclude that orofacial pain of cardiac origin is a heterotopic pain. The differential diagnosis of pain of odontogenic origin (dental and periodontal) must always be taken into account with pain of non-odontogenic origin (muscle pain, psychogenic, neuronal, cardiac, sinus and neurovascular) in order to avoid diagnostic errors in the dental practice as well as performing unnecessary treatments. Orofacial pain of cardiac origin is a bilateral pain, mainly located in the mandible and throat. It can irradiate to other craniofacial structures and also to more common areas such as the arms, shoulders and chest. It is paroxystic and severe, according to the clinical cases described in the articles consulted, but less intense that toothache and, additionally, less intense at more atypical areas of location of cardiac pain. It is not always bilateral and in the two cases presented it is not referred as especially intense.

The only comparative study between the quality of dental pain and heart pain, emphasizes that dental pain presents characteristics pulsating and stinging compared with cardiac pain described as burning and oppressive. The onset of the pain is usually spontaneous and can be triggered after performing physical exercise. It usually remains unchanged by movement or oral stimuli and is usually alleviated with adequate cardiac treatment.

When suspecting orofacial pain of cardiac origin, it is the dentist’s obligation to refer the patient to the cardiologist, with a detailed report of the tests performed.
